# Phenolic-Compound-Extraction Systems for Fruit and Vegetable Samples

**DOI:** 10.3390/molecules15128813

**Published:** 2010-12-03

**Authors:** Patricia Garcia-Salas, Aranzazu Morales-Soto, Antonio Segura-Carretero, Alberto Fernández-Gutiérrez

**Affiliations:** 1 Department of Analytical Chemistry, University of Granada, E-18071 Granada, Spain; 2 Functional Food Research and Development Centre, Health Science Technological Park, E-18100 Granada, Spain

**Keywords:** phenolic compounds, liquid-liquid extraction, solid-phase extraction, supercritical fluid extraction, pressurized liquid extraction, microwave-assisted extraction, ultrasound-assisted extraction

## Abstract

This paper reviews the phenolic-compound-extraction systems used to analyse fruit and vegetable samples over the last 10 years. Phenolic compounds are naturally occurring antioxidants, usually found in fruits and vegetables. Sample preparation for analytical studies is necessary to determine the polyphenolic composition in these matrices. The most widely used extraction system is liquid-liquid extraction (LLE), which is an inexpensive method since it involves the use of organic solvents, but it requires long extraction times, giving rise to possible extract degradation. Likewise, solid-phase extraction (SPE) can be used in liquid samples. Modern techniques, which have been replacing conventional ones, include: supercritical fluid extraction (SFE), pressurized liquid extraction (PLE), microwave-assisted extraction (MAE) and ultrasound-assisted extraction (UAE). These alternative techniques reduce considerably the use of solvents and accelerate the extraction process.

## 1. Introduction

Today’s society is characterized by having many unhealthy dietary habits. Not only snacking but also the inadequate intake of healthy foods triggers a major dietary imbalance, this being a major cause of chronic diseases such as obesity, diabetes mellitus, cardiovascular disease, hypertension, stroke, and several types of cancer. Therefore, it is vital to ascertain the composition and nutritional value of these products. To prevent the above-mentioned diseases, epidemiological studies recommend the consumption of whole fruits, vegetables, and legumes [1,2].

In recent decades, fruit and vegetable consumption has attracted growing interest because many epidemiological and biochemical studies have consistently demonstrated a clear and significant positive association between intake of these natural food products, consumed regularly as part of the Mediterranean diet, and reduced rates of heart disease, common cancers, and other degenerative diseases, as well as aging. The protection that fruits and vegetables provide against these maladies has been attributed to the presence of several antioxidants, especially to antioxidative vitamins, including ascorbic acid (vitamin C), α-tocopherol (vitamin E) and β-carotene (provitamin A). Nevertheless, recent studies seem to indicate that (poly) phenolic substances are the main phytochemicals with antioxidant properties found in higher plants [3,4,5].

Polyphenols, widely distributed in plants, contribute to fruit organoleptic and nutritive quality in terms of colour, taste, aroma, and flavour [6], also being involved in astringent and bitter tastes. It is known that, amongst other factors, such as maturity stage or light exposure, phenolic composition varies with the cultivar. In addition, the phenolic profile has already been revealed to be a useful parameter for the discrimination of the different fruit parts [7].

The intake of these compounds is an important health-protecting factor. These bioactive compounds retard or inhibit lipid autoxidation by acting as radical scavengers and, consequently, are essential antioxidants that protect against the propagation of the oxidative chain [8]. Evidence for their role in the prevention of degenerative diseases is emerging. Experimental studies on animal and human cell lines have demonstrated that polyphenols can play a role in preventing cancer and cardiovascular diseases, when taken daily in adequate amounts [9].

The determination of phenolic compounds in fruits, vegetables, and other foods has been of increasing interest in recent years [10]. Therefore, the objective of the present review is to show the classification of the polyphenolic compounds, taking into account different aspects related to these compounds. Moreover, our aim is to examine the various methods used for preparing and/or treating samples to determine the phenolic content in fruits and vegetables, including the different factors that affect the content in plant bioactive compounds, such as light, temperature, mineral nutrition, pathogens, mechanical damage, plant-growth regulators, and other factors [11].

## 2. Classification and Properties of Phenolic Compounds

Polyphenols have been a feature of plants since their early appearance. These compounds, also called secondary metabolites, are indeed crucial for many important functional aspects of plant life, including structural roles in different supportive or protective tissues, involvement in defence strategies, and signalling properties, particularly in the interactions between plants and their environment. Collectively, higher plants synthesise several thousand different known phenolic compounds, and the number of these which have been fully characterized is continually increasing [12].

The term "polyphenol" includes more than 8,000 compounds with great structural diversity (although each has at least one aromatic ring with one or more hydroxyl groups). They can be divided into 10 different classes depending on their basic chemical structure. Table 1 shows the main families of phenolic compounds, most of which are found in nature associated with mono- or polysaccharides (glycosides) or functional derivatives such as esters or methyl esters. Moreover, the main sources where phenolic compounds are found have been classified.

The most abundant polyphenols in the diet are phenolic acids (benzoic and cinnamic acids), and flavonoids (30 and 60% of the total, respectively) [13,14,15]. On the one hand, phenolic acids occur in different forms in plants, including aglycones (free phenolic acids), esters, glycosides, and/or bound complexes. These different forms of phenolic acids show variable suitability for different extraction conditions and vary in their susceptibility to degradation [16]. On the other hand, the common structure of flavonoids consists of two aromatic rings linked by three carbons that usually form an oxygenated heterocycle. In plants, flavonoids can be found as aglycones, although they are usually found as glycosides contributing to the colour (blue, scarlet, orange) of leaves, flowers, and fruits. Phenolic compounds are found not only in fruits and vegetables but also can be found in legumes, cereals, nuts, medicinal plants, spices, and beverages (e.g. tea, wine, and beer). Furthermore, flavonoids can be subdivided in 13 classes: chalcones, dihydrochalcone, auron, flavones, flavonols, dihydroflavonol, flavanones, flavanols (catechins), flavandioles or leucoanthocyanidins, anthocyanidins (its glycoside is called anthocyanin), isoflavononas, flavonoids, and condensed tannins or proanthocyanidins [13,17].

According to the epidemiological studies, the intake of phenolic compounds is inversely correlated with the risk of coronary heart disease [18,19]. In the human body, these phytochemicals are thought to provide health benefits by several mechanisms, including: (1) free-radical scavenging; (2) protection and regeneration of other dietary antioxidants (*i.e.* vitamin E); and (3) chelating of pro-oxidant metal ions. The species and levels of phenolic compounds vary dramatically among plants, and their different structures or levels are likely to have different functional properties [5,20]. Besides the general properties of the compounds, a number of polyphenolic compounds, especially catechins, have been found to be potent antioxidants and to be effective in preventing cancer [21] while tannins have been reported to exert other physiological effects; e.g. they can reduce blood pressure, accelerate blood clotting, lower serum-lipid levels, modulate immunoresponses and cause liver necrosis [22].

As mentioned above, it is impossible to separate the close relationship between the structure and properties of polyphenolics. The structure of phenolic compounds is a key determinant of their radical scavenging and metal-chelating activity. For example, in the case of phenolic acids, the antioxidant activity depends on the numbers and positions of the hydroxyl groups in relation to the carboxyl functional group. Thus, the antioxidant activity of phenolic acids increases the higher the degree of hydroxylation [14].

As a result, it is important to analyse the composition of phenolic compounds in fruits and vegetables before their health-promoting properties can be adequately studied. The analysis of phenolic compounds in plant samples is difficult because of the great variety of their structure and the lack of appropriate standards [5,20].

## 3. Extraction Systems for Phenolic Compounds

Extraction is one of the most important steps in sample pretreatment. Generally, it is a separation process where the distribution of the analyte (in this case, a phenolic compound) between two immiscible phases is made in order to arrive at the appropriate distribution coefficient [23]. The extraction procedure is sequential and systematically carried out using an aqueous organic solvent to extract phenolic compounds in fruit and vegetable samples. This traditional method is called liquid-liquid extraction (LLE) and different extraction solvents have been mentioned in the literature such as ethanol, acetone or methanol, or a mixture with water [16]. Soxhlet system is used to extract the lipidic fraction from food and other solid samples, using suitable solvents. Although it is not specific for phenolic compounds extraction, usually the extraction yields are compared to those obtained with another type of polyphenol extraction systems [24].

The ultimate goal of sample preparation is to eliminate or reduce potential matrix interferences [25]. The extraction must be performed with the most adequate solvent and under ideally predetermined analytical conditions of temperature and pH. Moreover, it is essential to take account the polyphenolic structure because these compounds may have multiple hydroxyl groups that can be conjugated to sugars, acids or alkyl groups. Thus, the polarities of phenolic compounds vary significantly and it is difficult to develop a single method for optimum extraction of all phenolic compounds. Hence, the optimisation of the extraction procedure is essential for an accurate assay of phenolic compounds from different food matrices.

In the end, the effort amounts to lowering costs and reducing sampling time during the above-mentioned conventional extraction. In any case, the extraction stage is extremely important, as its outcome will determine the release of analytes from the vegetable matrix into the medium, and this in turn will allow the quantitative determination of the extract [13].

For this reason, modern extraction and isolation techniques will be described as alternative techniques to considerably reduce solvent consumption and accelerate the extraction process. These modern techniques include: supercritical fluid extraction (SFE), pressurized liquid extraction (PLE), microwave-assisted extraction (MAE) and ultrasound-assisted extraction (UAE). These will be explained after the LLE description [26].

### 3.1. Liquid-Liquid Extraction (LLE)

Solubility of phenolics is governed by their chemical nature in the plant, which may vary from simple to very highly polymerized. Plant materials may contain varying quantities of phenolic acids, phenylpropanoids, anthocyanins, and tannins, among others. There is a possibility of interaction of phenolics with other plant components such as carbohydrates and proteins that may lead to the formation of complexes that may be quite insoluble. Likewise, the solubility of phenolics is affected by the polarity of solvent(s) used. Therefore, it is very difficult to develop an extraction procedure suitable for the extraction of all plant phenolics. The phenolic extracts from plant materials are always a diversified mixture of plant phenolics soluble in the solvent system used. Additional steps may be required to remove the unwanted phenolics and non-phenolic substances such as waxes, terpenes, fats, and chlorophylls [14,27].

The extraction methods for simple phenolic compounds (benzoic acids, benzoic aldehydes, cinnamic acids, and catechins) from solid or semi-solid materials have been focused on maceration using organic solvents. The current official analytical method for extracting phenolic compounds is liquid-liquid extraction (LLE) for liquid samples. This method requires expensive and hazardous organic solvents, which are undesirable for health and disposal reasons, and they require a long time per analysis, giving rise to possible degradations. The process of degradation can be triggered both by external and internal factors. Light, together with air and temperature, are the most important factors that facilitate degradation reactions. The extraction temperature usually needs to be high in order to minimise the duration of the process. For these reasons, these traditional extraction sample methods have been replaced by other methodologies which are more sensitive, selective, fast, and environmentally friendly [4,28]. In any case, LLE is still used as the standard preconcentration step for phenol determination in water because it is a cheap and easy method.

Solvents, such as methanol, ethanol, propanol, acetone, ethyl acetate, and their combinations have also been used for the extraction of phenolics, often with different proportions of water. For example, phenolic compounds can be efficiently extracted from legumes using an ethanol/water (70:30 v:v) system (see Table 2) [36].

Generally, LLE is used at room temperature to avoid the degradation of phenolic compounds, but there are many studies such as Costa *et al.*, Aparicio-Fernández *et al.* or Magalhães *et al.* using temperatures around 20 to 40 °C. When hydrolysis of phenolic compounds is carried out, the temperature is usually 80–95 °C for acid hydrolysis or 45 °C for basic hydrolysis [2,16,20,37,38]. Otherwise, extraction times depend on several factors such as maceration time, centrifugation time or the time spent on the evaporation of solvents.

Anthocyanins are usually extracted from plant material with an acidified organic solvent, most commonly methanol. This solvent system destroys the cell membranes, simultaneously dissolves the anthocyanins, and stabilizes them. However, the acid may bring about changes in the native form of anthocyanins by breaking down their complexes with metals and co-pigments [14]. An example is described by Ross *et al.*, where aglycone forms of glycoside flavonoids are obtained by acid hydrolysis of the bean extracts, using a methanol/water (85:15 v:v) system [16].

### 3.2. Solid-Phase Extraction (SPE)

Solid-phase extraction (SPE) is an increasingly useful sample-preparation technique. With SPE, many of the problems associated with liquid-liquid extraction, such as incomplete phase separations, less-than-quantitative recoveries, use and disposal of large and expensive quantities of organic solvents, can be avoided, although the cost of the equipment required for SPE is higher than for LLE. This technique is used most often to prepare liquid samples and extract semivolatile or nonvolatile analytes, but can also be used with solids that are pre-extracted into solvents. They are available in a wide variety of chemistries, adsorbents, and sizes so that it is necessary to select the most suitable product for each application and sample. For phenolic determination in grapes or wines and other beverages, different solid phases have been tested for SPE. Polymers of styrene-divinylbenzene provided good results, while C_18_-based phases afforded less satisfactory results for polar phenolics [10]. The particular case of phenolic extraction from olive-oil samples has been extensively studied. It is well known that the C_18_ phase is less suitable for the isolation of polar components from a nonpolar matrix than is the normal-phase SPE [27].

### 3.3. Supercritical Fluid Extraction (SFE)

Usually, phenolic compounds are extracted from plant samples by SPE coupled with other techniques, such as supercritical fluid extraction (SFE). SFE is a relatively recent technique which presents various advantages over traditional methods, such as the use of low temperatures and reduced energy consumption and high product quality due to the absence of solvents in the solute phase. However, this technique is limited to compounds of low or medium polarity. The literature offers descriptions of extraction methods for polyphenols by SFE, the main characteristics of which are the need for high percentages of organic modifiers; this usually means that the process takes place under subcritical conditions.

Supercritical carbon dioxide (SC-CO_2_) is the most widely used solvent for SFE due to its particular characteristics, such as moderate critical conditions (31.1 °C and 73.8 MPa) and ready availability. It is also nontoxic, inflammable and chemically stable. However, SFE using CO_2_ as the extracting solvent is of no use for phenolic compounds because of the low polarity of CO_2_ in comparison to most phenols [4,39].

Generally, for this extraction procedure, several steps are followed: samples are loaded onto the sorbent of the SPE cartridge, which is inserted into the SPE/SFE extraction cell. The supercritical fluid used can be carbon dioxide, which must go through the SPE cartridge filled with the hydrolysed sample. Thus, analytes (phenolic compounds) are quantitatively trapped by a trapping solvent (for example, methanol) at laboratory temperature (the trapping solvent is cooled naturally during the extraction by the expansion of CO_2_). Finally, the extracts are evaporated to dryness, dissolved in the mobile phase, and injected directly into the HPLC/ESI-MS system [28].

Castro-Vargas *et al.* compared different extraction systems for guava seed samples, the results of which are presented below. The yield of the SFE process in terms of phenolic fraction is also lower than the value achieved by Soxhlet extraction with ethanol (SE-EtOH), although the total extraction yields for SFE with CO_2_/EtOH are typically higher. This behaviour is explained by the non-polar characteristic of the carbon dioxide, which increases the extraction of low-polarity compounds, compared with polar ones (found particularly in the phenolic fraction) [39].

In SFE the yield results (phenolic and total) increase directly with solvent polarity and the use of EtOH as a co-solvent is particularly useful to enhance the phenolic fraction yield. At constant temperature, the rise in pressure increases the yield due to density enhancement. At constant pressure, the phenolic and the total yield decrease with rising temperatures due to the solvent density reduction. Lastly, it bears mentioning that SFE is of enormous interest today, with more than 200 references in the literature dealing with this topic in the last two years (2007-2009). The range of applications of SFE includes not only its use in sample preparation but also new and recent advances in different areas such as pharmaceutical, environmental science, and food science. With regard to the present results, readers are encouraged to treat the information as a tool to develop new processes at the laboratory and pilot scale, to discover new ways for sample preparation, to learn how to deal with SFE optimisation and, certainly, to be able to develop emerging technologies that can fulfil the requirements of environmentally clean processes [40].

### 3.4. Pressurized Liquid Extraction (PLE)

Pressurized liquid extraction (PLE) uses organic solvents at high pressures and temperatures above their normal boiling point. It is the newer modern method for isolation of analytes from solid samples [26]. In general, with PLE, a solid sample is packed into a stainless steel extraction cell and extracted with a suitable solvent under high temperatures (40–200 °C) and pressure (500-3000 p.s.i.) for short periods of time (5–15 min). The sample extract is purged into a collection vial with the aid of a compressed gas.

The procedure described by Alonso-Salces *et al.*, is based on polyphenol extraction in apple samples. Previously, freeze-dried samples are mixed with diatomaceous earth as a dispersion agent in order to reduce the solvent volume used for the extraction. The extracts are filtered, evaporated to dryness, reconstituted in methanol-aqueous hydrochloric acid 0.1% (30:70 v/v) and filtered again prior to injection into the HPLC system. These authors also examined different parameters such as percentages of methanol in the solvent, temperature, pressure, and static extraction time.

In Luthria *et al.*, all extractions were carried out with either one or two solvent mixtures, ethanol-water (50:50, v/v) and/or acetone-water (50:50, v/v), using a pressurized liquid extractor [25].

According to Liazid *et al.*, PLE has been shown to be effective as a method for extracting polyphenols, while rapid methods, taking 10 min, have been developed that use high temperatures (150 °C) to accelerate the process [4].

Briefly, Dobiáš *et al.* developed a new modern method for isolating analytes from solid samples, based on pressurised fluid extraction (PFE). In this case, the extraction process is carried out at higher temperature and higher pressure and the main advantages of this method involve low solvent consumption and a short extraction times [23].

### 3.5. Microwave-Assisted Extraction (MAE)

Microwave technology is commonly known for its use as heat treatment. For example, it is used as a heat process for commercial fruit products to achieve a fast but mild pasteurization of these products. At the same time, the use of microwaves serves to determine the stability of total polyphenol content after the treatment. As Picouet *et al.* conclude, significant losses occur during storage until the decrease of polyphenolic content is finished [41]. This technology is also used to speed up the drying process in wine and fresh grape samples, improving their pre-treatment and being a useful protocol to examine phenolic compounds [42].

Recently, microwave-assisted extraction (MAE), also called microwave-assisted process (MAP), has been applied in the development of extraction methods for organic compounds from soil, sediment, seed, and food matrices. These studies show that the extraction is more effective when microwave energy is used. The study by Sutivisedsak *et al.* demonstrates the utility of microwave-assisted extraction in determining the total phenolic contents of eight common bean types, using the Folin-Ciocalteau colorimetric method.

As occurs with SFE and PLE systems, MAE makes it possible to perform extractions in the absence of light. Phenolic compounds are very sensitive to this factor, giving these techniques a great advantage. This is important because, for example, resveratrol can be found in two isomeric forms (its *cis* and *trans* configurations), but only one of these, *trans*-resveratrol, presents biological activity. Light can catalyse the transformation from the active to the inactive form. In addition, the short extraction times that these techniques present (less than 1 h) reduce the adverse effects of enzymatic activity. Another important factor to be taken into account in the MAE is the temperature of the extraction. According to Liazid *et al.*, there is a clear relationship between the chemical structure and the stability of phenolic compounds that are studied under different conditions of MAE. Moreover, it has been shown that those that have a greater number of hydroxyl-type substituents are more easily degraded under these temperature conditions [4,43].

The main advantage of MAE is the possibility that several samples could be simultaneously extracted quicker than with Soxhlet extraction, and that similar recoveries to those of SFE were achieved. However, care must be taken when working with flammable solvents or in the case of samples that contain constituents which couple strongly with microwave radiation to cause a rapid rise in temperature and thereby lead to potentially hazardous situations [44].

### 3.6. Ultrasound-Assisted Extraction (UAE)

Ultrasonic radiation is a powerful aid in accelerating various steps of the analytical process. This energy is of great help in the pre-treatment of solid samples as it facilitates and speeds up operations such as the extraction of organic and inorganic compounds, homogenization, and various others. Ultrasound-assisted leaching is an effective way to extract analytes from different matrices in shorter times than with other extraction techniques [23]. For example, ultrasound-assisted systems have been widely used to extract capsaicinoids in hot peppers [45].

Ultrasonic extraction (USE) is considered one of the simplest extraction techniques because it is easy to perform in common laboratory equipment (*i.e.* ultrasonic bath). In this method, the crushed sample is mixed with the suitable solvent and placed into the ultrasonic bath, where the working temperature and extraction time are set [26].

The application of ultrasound-assisted extraction (UAE) in food-processing technology is of interest for facilitating the extraction of components from plant materials. The higher yield achieved in these UAE processes is of major interest from an industrial standpoint, since the technology is an add-on step to the existing process with minimum alteration, application in aqueous extraction where organic solvents can be replaced with solvents generally recognised as safe (GRAS), reduction in solvent usage, and shorter extraction time. The use of ultrasonic means for extraction purposes in high-cost raw materials is an economical alternative to traditional extraction processes, this being a demand by industry for a sustainable development.

Ultrasound can enhance existing extraction processes and enable new commercial extraction opportunities and processes. The main targets have been polyphenols and carotenoids and in both aqueous and solvent extraction systems. The ultrasound extraction trials have demonstrated improvements in extraction yield ranging from 6 to 35% [46].

Many studies have examined the stability of the analytes during ultrasound-assisted process. Herrero *et al.* evaluated the phenolic-compound decomposition when phenolics were subjected to solid-liquid, subcritical water or microwave-assisted extraction, and sonication was performed in order to assess the type of energy that provides a lower degradation of the analytes. The method was applied to two types of strawberries in order to demonstrate the applicability of the proposed method, which is much faster and results in less analyte degradation than do others [23].

Therefore, in recent years it has been shown that UAE offers lower phenolic compound recovery when compared to pressurized hot-water extraction methods. Vilkhu *et al.* proposed supercritical carbon dioxide extraction as a better method than ultrasound-assisted extraction of polyphenolic compounds from grape seeds. It was believed that the lower catechin (used as a measure of phenolic content) recovery from the ultrasound method could be due to the insufficient power of the solvent used (aqueous methanol) or due to the degradation of samples during extraction process. These authors focused on the efficiency of supercritical fluid extraction (SFE) rather than other methods used in the experiment. The results of catechin recovery using different extraction methods compared to a control (solvent extraction only) was not available and, consequently, it was not possible to determine whether ultrasound treatment (although having a lower recovery compared to SFE method) contributed to the increase in catechin recovery relative to a control. Most importantly, though, the frequency of ultrasound and other extraction conditions (e.g. temperature) was not stated, so that it is not known whether suitable frequencies or application conditions were used [46].

## 4. Conclusions

In this review, the advantages and disadvantages of different extraction systems for phenolic compounds are discussed. The most widely used extraction system is liquid-liquid extraction (LLE), which is an inexpensive method, since it involves the use of organic solvents, but it involves long extraction times, which give rise to possible degradations. Consequently, new techniques such as SFE, SPE, PLE, MAE, and UAE have been developed.

Normally, extraction efficiency increases at higher extraction temperatures, but the working temperature affects the stability of the phenolic compounds, which also depends on their chemical structure. Thus, factors that influence the extraction processes (temperature, polyphenolic structure, pressure, sample characteristics, and other factors) are discussed using examples.

## Figures and Tables

**Table 1:** Classification of families of phenolic compounds.

*Carbon numbers*	*Class*	*Basic structure*	*Sources*
C_6_	Simple phenols	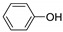	
	Benzoquinones	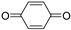	
C_6_-C_1_	Benzoic acid	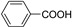	Cranberry, cereals
C_6_-C_2_	Acetophenones	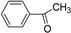	Apple, apricot, banana, cauliflower
	Phenylacetic acid	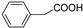	
C_6_-C_3_	Cinnamic acid	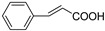	Carrot, citrus, tomato, spinach, peaches, cereal, pears, eggplant
	Phenylpropene	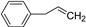	
	Coumarins	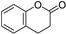	Carrot, celery, citrus, parsley
	Chromones	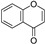	
C_6_-C_4_	Naphthoquinones	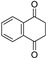	Nuts
C_6_-C_1_-C_6_	Xanthones	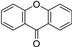	Mango, Mangosteen
C_6_-C_2_-C_6_	Stilbenes	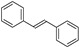	Grapes
	Anthraquinones	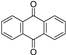	
C_6_-C_3_-C_6_	Flavonoids	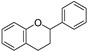	Widely distributed
(C_6_-C_3_)_2_	Lignans, neolignans	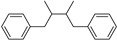 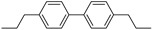	Sesame, rye, wheat, flax
(C_6_-C_1_)_n_	Hydrolysable tannins	Heterogeneous polymer composed of phenolic acids and simple sugars	Pomegranate, raspberry
(C_6_-C_3_)_n_	Lignins	Highly crosslinked aromatic polymer	

**Table 2:** LLE methods.

Sample	Reference	Solvent	Number of polyphenols identified
Bean (*Phaseolus vulgaris L*.)	[19]	Methanol (100%)	8
Bean (*Phaseolus vulgaris L*.)	[37]	Methanol/water (80:20 v:v), HCl 2N	6
Bean (*Phaseolus vulgaris L*.)	[11]	Acetonitrile HCl 0,1 N	3
Bean (*Phaseolus vulgaris L*.)	[2]	Methanol/water (80:20 v:v), HCl 2N	17
Bayberry (*Myrica rubra Sieb. et Zucc*)	[18]	Ethyl acetate	10
Artichoke (*Cynara Scolymus L*.)	[29]	Methanol/water (82:18 v:v)	3
Mustard greens (*Brassica juncea*)	[20]	Methanol/water (80:20 v:v)	3
Kale (*Brassica oleracea var. acephala*)	[20]	Methanol/water (80:20 v:v)	3
Okra (*Hibiscus esculentus L*.)	[20]	Methanol/water (80:20 v:v)	1
Potato (*Solanum tuberosum L*.)	[20]	Methanol/water (80:20 v:v)	2
Green Onion (*Allium fistulosum*)	[20]	Methanol/water (80:20 v:v)	1
Purslane (*Portulaca oleracea L*.)	[20]	Methanol/water (80:20 v:v)	3
Collard greens (*Brassica oleracea L*.)	[20]	Methanol/water (80:20 v:v)	2
Purple hull-peas (*Vigna unguiculata*)	[20]	Methanol/water (80:20 v:v)	1
Bean (*Phaseolus vulgaris L*.)	[30]	Acetone 80%	4
Bean (*Phaseolus vulgaris L*.)	[31]	Methanol/water (85:15 v:v), HCl 1M	7
Parsley flakes (*Petroselinum crispum L*.)	[25]	Methanol	1
Quince (*Cydonia oblonga L*.)	[5]	Methanol (100%)	18
Tree tomato (*Cyphomandra betacea L*.)	[1]	Acetone 70%	8
Naranjilla (*Solanum quitoense L*.)	[1]	Acetone 70%	2
Artichoke (*Cynara Scolymus L*.)	[32]	Methanol/water (50:50 v:v)	15
Garlic (*Allium sativum L*.)	[38]	Methanol/water (50:50 v:v)	2
Onion (*Allium cepa L*.)	[38]	Methanol/water (50:50 v:v)	2
Bean (*Phaseolus vulgaris L*.)	[16]	Methanol/water (85:15 v:v)	12
Papaya (*Carica papaya L*.)	[33]	Methanol (100%)	12
Eggplant (*Solanum melongena L*.)	[34]	Methanol/water (80:20 v:v)	18
Eggplant (*Solanum melongena L*.)	[17]	Methanol (100%)	4
Red lettuce (*Lactuca sativa L*.)	[17]	Methanol (100%)	4
Red onion (*Allium fistulosum L*.)	[17]	Methanol (100%)	10
Bean (*Phaseolus vulgaris L*.)	[17]	Methanol (100%)	9
Pistachio (*Pistacia vera L*.)	[17]	Methanol (100%)	2
Cucumber (*Cucumis sativus L*.)	[35]	DMSO	11

## References

[1] Mertz C., Gancel A.-L., Punata Z., Alter P., Dhuique-Mayer C., Vaillant F., Perez A.M., Ruales J., Prat P. (2009). Phenolic compounds, carotenoids and antioxidant activity of three tropical fruits. J. Food. Compos. Anal..

[2] Espinosa-Alonso L.G., Lygin A., Widholm J.M., Valverde M.E., Octavio Paredes-López O. (2006). Polyphenols in wild and weedy mexican common beans (Phaseolus vulgaris L.). J. Agric. Food Chem..

[3] Rodríguez-Medina I.C., Segura-Carretero A., Fernández-Gutiérrez A. (2009). Use of high-performance liquid chromatography with diode array detection coupled to electrospray-Qq-time-of-flight mass spectrometry for the direct characterization of the phenolic fraction in organic commercial juices. J. Chromatogr. A..

[4] Liazid A., Palma M., Brigui J., Barroso G.C. (2007). Investigation on phenolic compounds stability during microwave-assisted extraction. J. Chromatogr. A..

[5] Magalhães A.S., Silva B.M., Pereira J.A., Andrade P.B., Valentão P., Carvalho M. (2009). Protective effect of quince (Cydonia oblonga Miller) fruit against oxidative hemolysis of human erythrocytes. Food Chem. Toxicol..

[6] Serrano M., Zapata P.J., Castillo S., Guillén F., Martínez-Romero D., Valero D. (2010). Antioxidant and nutritive constituents during sweet pepper development and ripening are enhanced by nitrophenolate treatments. Food Chem..

[7] Ferreres F., Gomes D., Valentão P., Gonçalves R., Pio R., Alves E., Seabra R.M., Andrade P.B. (2009). Improved loquat (*Eriobotrya japonica Lindl*.) cultivars: variation of phenolics and antioxidative potential. Food Chem..

[8] Navarro J.M., Flores P., Garrido C., Martinez V. (2006). Changes in the contents of antioxidant compounds in pepper fruits at different ripening stages, as affected by salinity. Food Chem..

[9] Wijngaard H.H., Rößle C., Brunton N. (2009). A survey of Irish fruit and vegetable waste and by-products as a source of polyphenolic antioxidants. Food Chem..

[10] Palma M., Piñeiro Z., Barroso C.G. (2002). In-line pressurized-fluid extraction-solid-phase extraction for determining phenolic compounds in grapes. J. Chromatogr. A..

[11] Dinelli G., Bonetti A., Minelli M., Marotti I., Catizone P., Mazzanti A. (2006). Content of flavonols in italian bean (*Phaseolus vulgaris L*.) ecotypes. Food Chem..

[12] Boudet A.M. (2007). Evolution and current status of research in phenolic compounds. Phytochemistry.

[13] Escarpa A., Gonzalez M.C. (2008). An overview of analytical chemistry of phenolic compounds in foods. Crit. Rev. Anal. Chem..

[14] Naczk M., Shahidi F. (2006). Phenolics in cereals, fruits and vegetables: occurrence, extraction and analysis. J. Pharm. Biomed. Anal..

[15] Balasundram N., Sundram K., Samman S. (2006). Phenolic compounds in plants and agri-industrial by-products: Antioxidant activity, occurrence, and potential uses. Food Chem..

[16] Ross K.A., Beta T., Arntfield S.D. (2009). A comparative study on the phenolic acids identified and quantified in dry beans using HPLC as affected by different extraction and hydrolysis methods. Food. Chem..

[17] Wu X., Prior R.L. (2005). Identification and characterization of anthocyanins by high-performance liquid chromatography-electrospray ionization-tandem mass spectrometry in common foods in the United States: vegetables, nuts, and grains. J. Agr. Food Chem..

[18] Fang Z., Zhang M., Wang L. (2007). HPLC-DAD-ESI-MS analysis of phenolic compounds in bayberries (*Myrica rubra Sieb. et Zucc*.). Food Chem..

[19] Aparicio-Fernández X., Yousef G.G., Loarca-Pina G., de Mejia E., Lila M.A. (2005). Characterization of polyphenolics in the seed coat of black jamapa bean (Phaseolus vulgaris L.). J. Agr. Food Chem..

[20] Huang Z., Wang B., Eaves D.H., Shikany J.M., Pace R.D. (2007). Phenolic compound profile of selected vegetables frequently consumed by african americans in the southeast. US Food Chem..

[21] Costa R.M., Magalhães A.S., Pereira J.A., Andrade P.B., Valentão P., Carvalho M., Silva B.M. (2009). Evaluation of free radical-scavenging and antihemolytic activities of quince (*Cydonia oblonga*) leaf: a comparative study with green tea (*Camellia sinensis*). Food Chem. Toxicol..

[22] Muchuweti M., Ndhlala A.R., Kasiamhuru A. (2006). Analysis of phenolic compounds including tannins, gallotannins and flavanols of *Uapaca kirkiana* fruit. Food Chem..

[23] Dobiáš P., Pavlíková P., Adam M., Eisner A., Beňová B., Ventura K. (2010). Comparison of pressurised fluid and ultrasonic extraction methods for analysis of plant antioxidants and their antioxidant capacity. Cent. Eur. J. Chem..

[24] Arias M., Penichet I., Ysambertt F., Bauzab R., Zougaghc M., Ríos A. (2009). Fast supercritical fluid extraction of low- and high-density polyethylene additives: Comparison with conventional reflux and automatic Soxhlet extraction. J. Supercrit. Fluid..

[25] Luthria D.L. (2008). Influence of experimental conditions on the extraction of phenolic compounds from parsley (*Petroselinum crispum*) flakes using a pressurized liquid extractor. Food Chem..

[26] Klejdusa B., Kopecký J., Benesˇová L., Vaceka J. (2009). Solid-phase/supercritical-fluid extraction for liquid chromatography of phenolic compounds in freshwater microalgae and selected cyanobacterial species. J. Chromatogr. A..

[27] Gómez A.M., Carrasco A., Cañabate B., Segura A., Fernández A. (2005). Electrophoretic identification and quantitation of compounds in the polyphenolic fraction of extra-virgin olive oil. Electrophoresis.

[28] Mahugo C., Sosa Z., Torres M.E., Santana J.J. (2009). Methodologies for the extraction of phenolic compounds from environmental samples: new approaches. Molecules.

[29] Gil-Izquierdo A., Gil M.I., Conesa M.A., Ferreres F. (2001). The effect of storage temperatures on vitamin C and phenolics content of artichoke (*Cynara scolymus* L.) heads. Innov. Food Sci. Emerg..

[30] Karamac M., Amarowicz R. (2004). Antioxidant activity of phenolic fractions of white bean (*Phaseolus vulgaris*). J. Food Lipids..

[31] Laparra J.M., Glahn R.P., Miller D.D. (2008). Bioaccessibility of phenols in common beans (*Phaseolus vulgaris L*.) and iron (Fe) availability to caco-2 cells. J. Agric. Food Chem..

[32] Mulinacci N., Prucher D., Peruzzi M., Romani A., Pinelli P., Giaccherini C., Vincieri F.F. (2004). Commercial and laboratory extracts from artichoke leaves: estimation of caffeoyl esters and flavonoidic compounds content. J. Pharm. Biomed..

[33] Simirgiotis M.J., Caligari P.D.S., Schmeda-Hirschmann G. (2009). Identification of phenolic compounds from the fruits of the mountain papaya Vasconcellea pubescens A. DC. grown in Chile by liquid chromatography-UV detection-mass spectrometry. Food Chem..

[34] Singh A.P., Luthria D., Wilson T., Vorsa N., Singh V., Banuelos G.S., Pasakdee S. (2009). Polyphenols content and antioxidant capacity of eggplant pulp. Food Chem..

[35] Yaginuma S., Shiraishi T., Ohya H., Igarashi K. (2002). Polyphenol increases in safflower and cucumber seeding exposed to strong visible light with limited water. Biosci. Biotechnol. Biochem..

[36] Prati S., Baravelli V., Fabbri D., Schwarzinger C., Brandolini V., Maietti A., Tedeschi P., Benvenuti S., Macchia M., Marotti I., Bonetti A., Catizone P., Dinelli G. (2007). Composition and content of seed flavonoids in forage and grain legume crops. J. Sep. Sci..

[37] Diaz-Batalla L., Widholm J.M., Fahey G.C., Castaño-Tostado €., Paredes-López O. (2006). Chemical components with health implications in wild and cultivated mexican common bean seeds (*Phaseolus vulgaris* L.). J. Agric. Food Chem..

[38] Nuutila A.M., Puupponen-Pimia R., Aarni M., Oksman-Caldentey K.M. (2003). Comparison of antioxidant activities of onion and garlic extracts by inhibition of lipid peroxidation and radical scavenging activity. Food Chem..

[39] Castro-Vargas H.I., Rodríguez-Varela L.I., Ferreira S.R.S., Parada-Alfonso F. (2010). Extraction of phenolic fraction from guava seeds (*Psidium guajava L*.) using supercritical carbon dioxide and co-solvents. J. Supercrit. Fluid..

[40] Herrero M., Mendiola J.A., Cifuentes A., Ibáñez E. (2010). Review: Supercritical fluid extraction: recent advances and applications. J. Chromatogr. A..

[41] Picouet P.A., Landl A., Abadias M., Castellari M., Viñas I. (2009). Minimal processing of a Granny Smith apple purée by microwave heating. Innov. Food Sci. Emerg..

[42] Chu T.Y., Chang C.H., Liao Y.C., Chen Y.C. (2001). Microwave-accelerated derivatization processes for the determination of phenolic acids by gas chromatography-mass spectrometry. Talanta.

[43] Sutivisedsak N., Cheng H.N., Willett J.L., Lesch W.C., Tangsrud R.R., Biswas A. (2010). Microwave-assisted extraction of phenolics from bean (Phaseolus vulgaris L.). Food Res. Int..

[44] Jáuregui O., Galceran M.T. (2001). Handbook of Analytical Separations.

[45] Barbero G.F., Liazid A., Palma M., Barroso C.G. (2008). Ultrasound-assisted extraction of capsaicinoids from peppers. Talanta.

[46] Vilkhu K., Mawson R., Simons L., Bates D. (2008). Applications and opportunities for ultrasound assisted extraction in the food industry. Innov. Food Sci. Emerg..

